# Case report: complete pilot balloon detachment in a ventilator-dependent patient with high-risk tracheostomy exchange

**DOI:** 10.3389/fmed.2026.1994056

**Published:** 2026-10-21

**Authors:** Xinyu Lai, Yan Huang, Mingyu Hu, Lin Huang

**Affiliations:** 1 Intensive Care Unit, Xiangya Changde Hospital, Changde, Hunan, China; 2 Department of Nursing, Xiangya Hospital, Central South University, Changsha, Hunan, China

**Keywords:** airway management, bridge management, case report, cuff inflation-system failure, mechanical ventilation, pilot balloon, tracheostomy

## Abstract

**Background:**

Complete detachment of a pilot balloon and one-way valve eliminates the standard route for tracheostomy-cuff inflation and pressure measurement. Definitive tube exchange may be hazardous in selected patients.

**Case description:**

A 74-year-old ventilator-dependent woman with a long-term tracheostomy developed an audible upper-airway leak, a ventilator leak alarm, and reduced displayed tidal volume. Inspection showed complete loss of the pilot balloon and one-way valve, while the residual inflation line remained attached to the tube. Computed tomography documented post-tracheostomy changes but did not report peri-tracheostomy granulation. Otolaryngology bedside examination identified granulation tissue below the stoma and considered tube removal and exchange difficult, with risks of bleeding, aspiration, respiratory difficulty, and loss of the airway. After multidisciplinary assessment and informed shared decision-making, the existing tube was retained and an exceptional bridge manoeuvre was used to access the residual inflation-line lumen, inject approximately 3 mL of air, and occlude the line. Because the pilot balloon and one-way valve were absent, the standard connection for a cuff manometer no longer existed; intracuff pressure was not measured. The reported volume was case-specific, was not a target, and did not establish a safe cuff pressure, efficacy, or safety. Routine hourly observations near the event were not immediate pre-/post-intervention measurements and cannot demonstrate an intervention-related physiological effect. Intermittent leakage persisted, and the defective tube remained *in situ* at family-requested discharge.

**Conclusion:**

This case highlights recognition of cuff-system failure, specialist escalation, shared risk assessment, and surveillance when exchange is hazardous. This case-specific manoeuvre should not be interpreted as a recommended bedside procedure. Definitive management remains replacement of the defective tracheostomy tube whenever safely feasible.

## Introduction

Adults with tracheostomies require structured surveillance, trained responders, and coordinated multidisciplinary care; tracheostomy bundles and specialist teams can improve care processes and reduce adverse events (1, 2). In a patient receiving positive-pressure ventilation, the cuff helps establish a functional seal. Failure of the pilot balloon, one-way valve, or inflation line can therefore cause an audible leak, ventilator leak alarms, and loss of delivered tidal volume. Published reports of disrupted pilot-balloon systems mainly concern endotracheal tubes and describe temporary reconstruction of the pilot-balloon assembly when tube replacement is judged excessively hazardous (3).

The present case is distinct because the pilot balloon and one-way valve were completely detached from a tracheostomy tube in a ventilator-dependent patient, tube exchange was judged high risk after specialist airway assessment, and the bridge manoeuvre did not reconstruct either the one-way valve or the pilot-balloon assembly. Replacement of a tracheostomy tube with an irreparable cuff inflation system remains definitive management. In accordance with the CARE guideline (4), this report focuses on recognition, airway risk assessment, exceptional bridge management, and surveillance when immediate exchange was considered hazardous.

## Case description

A 74-year-old woman with chronic respiratory dysfunction and type II respiratory failure was managed in an intensive care unit in China and required long-term tracheostomy and mechanical ventilation. Her comorbidities included coronary artery disease, heart failure, Parkinson disease, sequelae of cerebral infarction, diabetes, and hypertension. She underwent tracheostomy on 10 March 2021 and subsequently had multiple admissions to the same hospital. The tube involved in this event had last been replaced on 18 August 2023. It was a cuffed percutaneous dilatational tracheostomy tube (PW16-S-70; Shanghai QA Medical Instrument Co., Ltd., Shanghai, China) with a 7.0-mm internal diameter and a 10.5-mm external diameter. The event occurred during her ninth hospitalisation. The chronology is shown in Figure 1.

**Figure 1:**
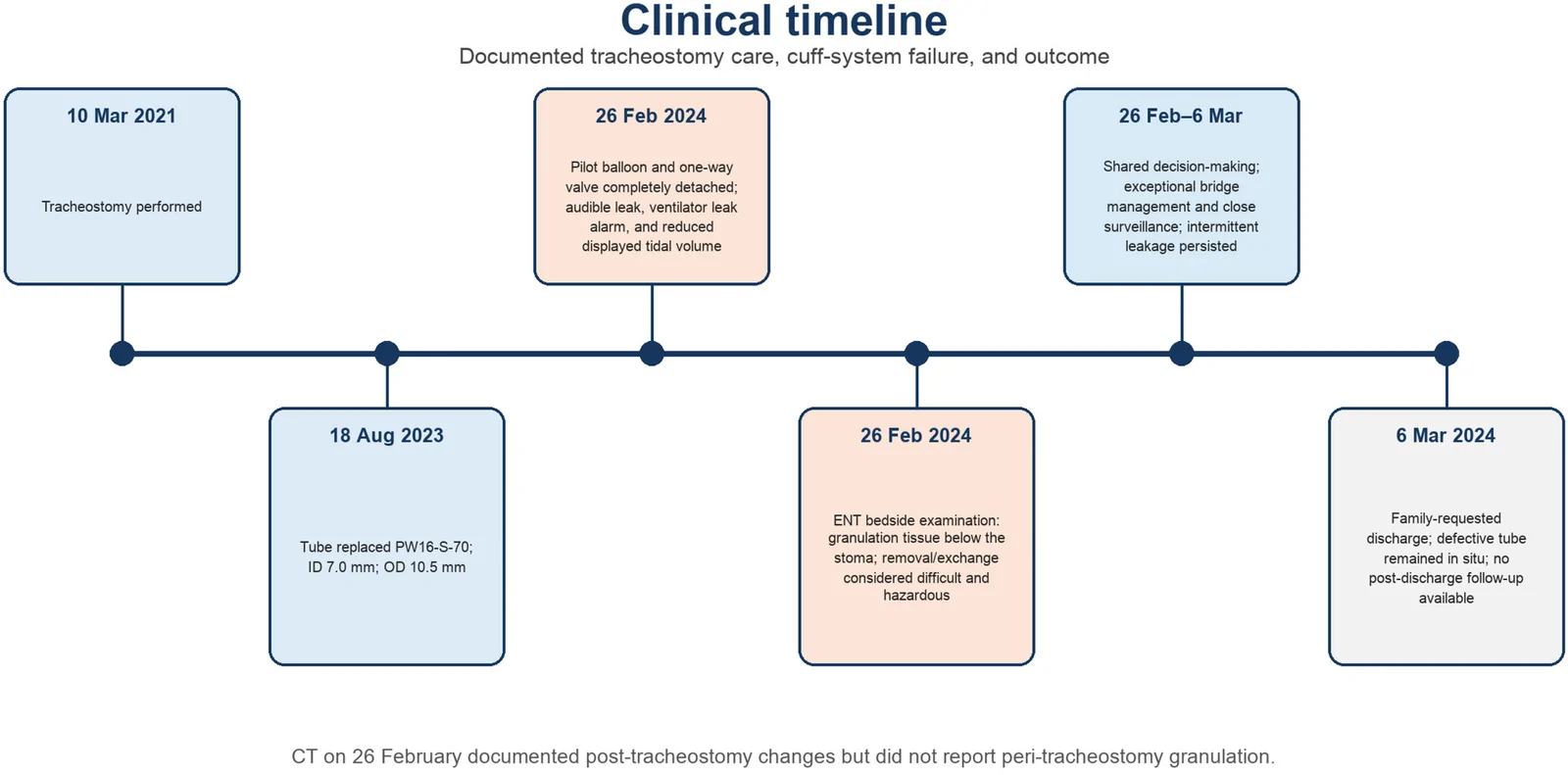
Clinical timeline of documented tracheostomy care, complete pilot-balloon and one-way-valve detachment, specialist airway assessment, bridge management, and outcome. CT, computed tomography; ENT, ear, nose and throat; ID, internal diameter; OD, external diameter.

On the morning of 26 February 2024, the bedside intensive care nurse heard a new air leak from the upper airway while the patient was receiving mechanical ventilation. A ventilator leak alarm occurred concurrently, and displayed tidal volume fell. The nurse assessed patient, circuit, and airway causes and then inspected the external tracheostomy assembly. The blue pilot balloon, including its Luer one-way valve, had completely detached from the blue cuff inflation line and was no longer present. A residual inflation-line segment remained connected to the tracheostomy tube. The concordant findings supported cuff inflation-system failure rather than an isolated ventilator malfunction.

## Diagnostic assessment and airway risk evaluation

Definitive tube exchange was considered. Chest computed tomography on 26 February documented post-tracheostomy changes, bronchitis, bilateral pneumonia similar to earlier imaging, bilateral pleural effusions, and adjacent incomplete lung expansion; it did not report peri-tracheostomy granulation tissue or airway narrowing. During bedside examination, the otolaryngology team observed granulation tissue below the tracheostomy stoma; the surrounding area was clean and without obvious secretions. The team considered difficult removal potentially related to granulation tissue and advised that exchange could cause bleeding, aspiration, respiratory difficulty, or loss of the airway. No bronchoscopy was documented in relation to this event; airway risk was assessed clinically by the otolaryngology team.

The intensive care and otolaryngology teams considered both the consequences of continued cuff-system failure and the hazards of immediate exchange. They explained the specialist findings and the risks of exchange and non-exchange to the patient's family. After the family indicated understanding, the treating team and family reached an informed shared decision to defer exchange, retain the existing tube, continue ventilatory support, and use exceptional bridge management with close observation. Family preference alone was not treated as the clinical basis for withholding definitive management; the decision reflected the team's assessment that bridge management was temporarily acceptable while immediate exchange carried substantial case-specific airway risk.

## Therapeutic intervention

To obtain temporary access to the cuff, the nurse attached the needle supplied with a 1-mL syringe to a 5-mL syringe and inserted the needle into the lumen of the residual inflation line rather than through its wall. Air was injected incrementally. After approximately 3 mL had been delivered, the audible leak and ventilator leak alarm were no longer present at that observation. The needle was withdrawn, and the residual line was occluded with a straight haemostatic clamp to limit air escape. The pilot balloon and one-way valve had completely detached; therefore, the standard connection used for a cuff manometer no longer existed and intracuff pressure could not be measured by the usual method. The approximately 3-mL volume was used only in this individual event. It was not a target volume, is not generalisable, cannot demonstrate that a safe intracuff pressure was achieved, and does not establish efficacy or safety. The manoeuvre did not constitute repair or restoration of normal cuff function. This case-specific manoeuvre should not be interpreted as a recommended bedside procedure. Definitive management remains replacement of the defective tracheostomy tube whenever safely feasible.

The chart contained routine hourly observations rather than measurements acquired specifically immediately before and after the bridge manoeuvre. At 07:00, oxygen saturation was 99%, heart rate 79 beats/min, respiratory rate 19 breaths/min, blood pressure 112/46 mmHg (mean 66 mmHg), peak airway pressure approximately 21 cmH₂O, mean airway pressure approximately 11 cmH₂O, and displayed tidal volume approximately 491 mL. The 08:00 ventilator row recorded P-SIMV mode, positive end-expiratory pressure 5 cmH₂O, inspired oxygen fraction 50%, peak airway pressure approximately 20 cmH₂O, mean airway pressure approximately 11 cmH₂O, and displayed tidal volume approximately 466 mL. At 09:00, oxygen saturation was 100%, heart rate 65 beats/min, respiratory rate 14 breaths/min, and blood pressure 103/55 mmHg (mean 70 mmHg). Delivered and exhaled tidal volumes were not recorded as paired values, and minute ventilation was unavailable. Because these were routine hourly observations and were not temporally designed as immediate pre-/post-intervention measurements, they cannot establish an intervention-related physiological effect. Arterial blood gas measurements temporally linked to the cuff-system failure and bridge manoeuvre were not available. The case-specific decision framework is shown in Figure 2.

**Figure 2:**
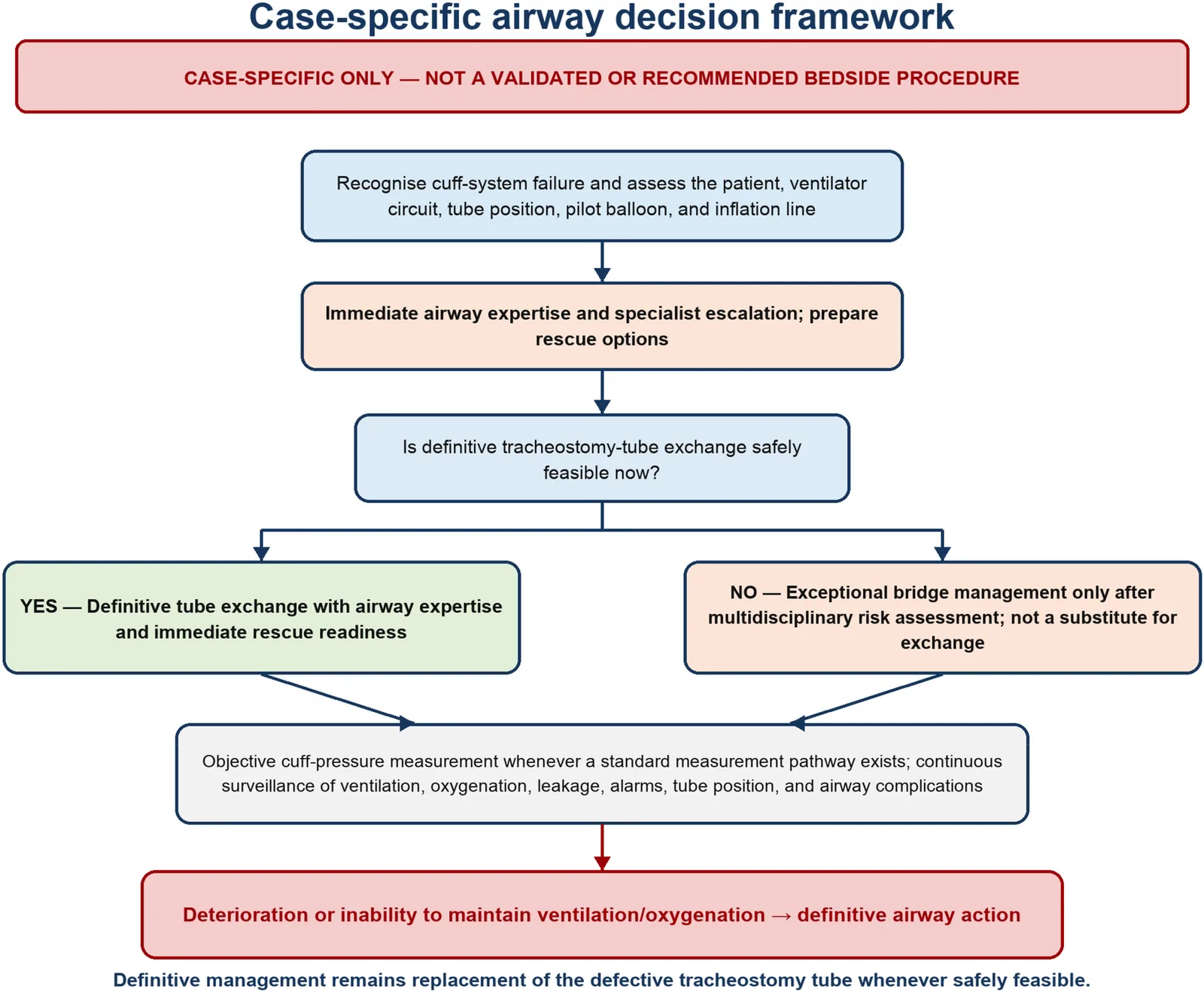
Case-specific airway decision framework after suspected tracheostomy cuff inflation-system failure. The framework emphasises patient and equipment assessment, immediate airway expertise, specialist escalation, definitive tube exchange whenever safely feasible, exceptional bridge management only after multidisciplinary risk assessment, objective cuff-pressure measurement whenever a standard measurement pathway exists, continuous surveillance, and definitive airway action if deterioration occurs. It is not a validated or recommended bedside procedure.

## Follow-up and outcomes

Contemporaneous records documented continued ventilator dependence and recurrent air leakage through discharge. On 29 February, the patient remained on P-SIMV ventilation with a positive end-expiratory pressure of 5 cmH₂O and an inspired oxygen fraction of 50%; oxygen saturation was 100%, and air leakage around the tracheostomy was still recorded. At discharge on 6 March, she remained on P-SIMV ventilation with a positive end-expiratory pressure of 6 cmH₂O and an inspired oxygen fraction of 60%. Air leakage was described as obvious, with episodic tachypnoea and continued sputum; temperature was 36.5 °C, heart rate 77 beats/min, respiratory rate 28 breaths/min, blood pressure 108/63 mmHg, and oxygen saturation 99%. These observations describe clinical status and do not establish an effect of the bridge manoeuvre.

The nursing team retrospectively recalled that cuff reinflation was commonly required approximately two to four times daily and that approximately 3 mL of air was usually used. These frequency and volume estimates were not contemporaneous quantified nursing-record data. Intermittent leakage persisted, indicating that normal cuff function had not been restored and that a durable seal had not been achieved.

Chest computed tomography documented bilateral pneumonia similar to earlier imaging; the available record did not attribute the pneumonia to the cuff-system failure or bridge manoeuvre. No contemporaneous event-specific record of aspiration, pneumothorax, subcutaneous emphysema, or airway bleeding was available. Absence of such documentation cannot establish that these complications did not occur. The defective tracheostomy tube was not removed during the hospitalisation and remained *in situ* at discharge. Consequently, the device was not available to the authors for post-event inspection or engineering analysis, and the mechanism of pilot-balloon detachment could not be determined.

The family requested discharge and completed the documented discharge process after the team explained the risks during transfer and after discharge. No post-discharge clinical follow-up, including any reliable documentation of later outcome, was available to the authors.

## Discussion

### Why the device failure mattered

Complete loss of the pilot balloon and one-way valve removed both the intended route for cuff inflation and the standard connection for pressure measurement in a patient dependent on positive-pressure ventilation. The simultaneous audible leak, leak alarm, reduced displayed tidal volume, and absent external components provided a coherent pattern that prompted inspection of the inflation system. Laboratory work has shown that selected disrupted endotracheal-tube pilot-balloon assemblies can be temporarily reconstructed with an intraluminal catheter when replacement is hazardous (3). This tracheostomy case involved complete external-component detachment and no reconstruction of the one-way valve or pilot balloon. Access to the residual line followed by mechanical occlusion therefore differed materially from previously described endotracheal-tube reconstruction.

### Why immediate tube exchange was hazardous

Tube exchange remained the definitive response to the irreparable cuff inflation system. The anatomical concern in this case came from otolaryngology bedside examination, not from a CT diagnosis of granulation tissue. Granulation was observed below the stoma, removal of the existing tube was difficult, and the specialist assessment identified risks of bleeding, aspiration, respiratory difficulty, and loss of the airway. Long-term tracheostomy in ventilator-dependent patients can be complicated by granulation tissue and obstruction (5), while difficult exchange may be associated with false passage, subcutaneous emphysema, pneumothorax, or airway loss (6, 7). The decision to defer exchange was consequently made through informed shared decision-making after clinical airway-risk assessment, with continued readiness to escalate if ventilation or oxygenation could not be maintained.

### Safety boundaries and clinical lessons

This case does not establish that the bridge manoeuvre is safe or effective. Without manometry, a fixed air volume cannot be translated reliably into intracuff pressure across different tube designs and anatomical conditions (8–10). Potential hazards include uncontrolled intracuff pressure, overinflation, cuff rupture, inflation-line damage, inadvertent air loss, loss of positive-pressure ventilation, and airway injury. Disappearance of a leak alarm cannot verify a safe cuff pressure, and recurrent leakage in this case showed that a durable seal had not been achieved.

The transferable lesson is the decision process: integrate patient findings with ventilator information and physical inspection; seek early airway expertise; assess whether definitive exchange can be performed safely; use objective cuff-pressure measurement whenever the standard pathway is available; and trigger definitive airway action if ventilation or oxygenation deteriorates. These functions are consistent with multidisciplinary tracheostomy care and emergency guidance (1, 2, 6). Figure 2 presents this case-specific framework without depicting the exceptional needle-and-clamp manoeuvre as a standard option.

## Limitations

This report describes a single retrospective case. The standard cuff-manometer connection was absent and intracuff pressure was not measured. Immediate paired pre-/post-intervention ventilator measurements, minute ventilation, event-linked arterial blood gases, and complete complication surveillance were unavailable. Reinflation frequency and approximate volume were based on retrospective staff recollection rather than contemporaneous quantified records. Intermittent leakage persisted. The tube remained *in situ*, precluding device inspection and engineering analysis, and the detachment mechanism could not be determined. No post-discharge follow-up was available. The case cannot establish effectiveness, reproducibility, generalisability, or safety.

## Conclusion

Complete detachment of the pilot balloon and one-way valve in this ventilator-dependent patient required prompt recognition, specialist airway assessment, informed shared decision-making, and close surveillance while immediate exchange was considered hazardous. The bridge manoeuvre neither restored the one-way valve nor provided objective control of intracuff pressure, and leakage persisted. It should not be interpreted as a recommended bedside procedure. Definitive management remains replacement of the defective tracheostomy tube whenever safely feasible.

## Patient perspective

A patient or family perspective on the bridge-management experience was not available in the source information supplied for this report.

## Data Availability

The original contributions presented in this case report are included in the article. Further inquiries can be directed to the corresponding author.
